# Measuring teacher identity of physicians – a validation study of a questionnaire instrument

**DOI:** 10.1080/10872981.2024.2333618

**Published:** 2024-03-25

**Authors:** Ann-Kathrin Schindler, Mareike Schimmel, Melissa Oezsoy, Thomas Rotthoff

**Affiliations:** a Medical Didactics and Education Research; DEMEDA (Department of Medical Education); Medical Faculty, University of Augsburg, Augsburg, Germany; b Pediatrics and Adolescent Medicine, University Hospital Augsburg, Augsburg, Germany; c Education and Educational Psychology, Faculty of Psychology and Educational Science, University of Munich (LMU), Munich, Germany

**Keywords:** Teacher identity, role of teacher, questionnaire instrument, factor analysis, faculty development

## Abstract

**Background:**

Teacher identity is defined as a continuum of a person’s self-conviction (‘Identity is something I have’) and a context-dependent action (‘Identity is something I do in a context’) (Lankveld et al. 2021). It has been identified a relevant contributor to physicians’ teaching commitment. In this study, we further improve the currently only existing questionnaire instrument (37 items) measuring physicians’ teacher identity.

**Methods:**

Survey data on 147 clinicians at a German university hospital were (1) analyzed by confirmatory factor analysis (CFA). We tested (a) the model fits of the originally suggested scales and (b) potential for improvement of model fits by item reduction. As this could not reveal satisfactory fits for all scales, we (2) applied a principal axis factoring as an exploratory approach. Last, we combined findings from (1) and (2) with a theoretical item content discussion and suggest (3) reassembled scales which were again checked using CFA.

**Findings:**

(1a) Two scales from the original instrument were successfully confirmed. (1b) Some scales benefited from item reduction. (2) The exploratory analysis identified three factors that explained at least 5% variance. (3) By integrating confirmatory and exploratory findings with a content analysis of the items, we propose a partially rearranged questionnaire instrument, comprising seven scales: (1) Feeling intrinsic satisfaction from teaching; (2) Feeling responsibility to teach; (3) Exchange of teaching experience; (4) Identification and enjoyment of the teaching role; (5) Development of teaching; (6) Teaching self-concept of ability; (7) Desired rewards for teaching. Four items were kept as single items.

**Conclusion:**

We suggest that when assessing teacher identity in physicians, all items should be constructed to allow for responses, even from physicians who are presently not actively involved in teaching. The scales benefited from categorizing items based on the continuum of teacher identity as outlined by van Lankveld et al. (2021).

## Introduction

Teacher identity is defined as a continuum of a person’s self-conviction (‘Identity is something I have’) and a context-dependent action (‘Identity is something I do in a context’) [1], p. 125). It has been identified as a contributor to physicians’ teaching commitment and quality, job satisfaction and reduced teaching-related burdens [2–4].

Measuring teacher identity in a reliable, factorial valid and economical way can help to provide a better way to define didactical and organisational faculty development offers based on medical teachers’ needs and explicate the relevance of the topic. A review [5] on teacher identity in the university context identified 59 studies; of these, 57 applied qualitative methods, such as interviews or focus group discussions to assess teacher identity. Qualitative assessments of teacher identity have the power to discover its various constituents but lack an economical application to repeated measurements or large-scale assessments [6], for example, when questioning physicians in an entire hospital. Hanna et al. [6] identified the only existing questionnaire instrument measuring physicians’ teacher identity, developed by Starr et al. [7], including 37 items nested in nine scales designed from previously conducted interviews [8]. In their initial testing on a sample of *N* = 127 primary care physicians with varying levels of teaching experience, Cronbach’s α ranged from .51 to .88 (see Table 1). Sherman et al. [9] confirmed the lack of internal consistency of some scales (see Table 1). In their measurement, the sample consisted of *N* = 297 physicians who transferred from medical school to residency.

**Table 1.** Internal consistency of original scales in previous studies, results of CFAs and internal consistency of original scales (RQ1a) and scales after item reduction (RQ1b) in the current study.

		Internal consistency in previous studies			Fit indices^1^ and internal consistency^2^ of the original scales with 37 items (RQ1a)						Fit indices^1^ and internal consistency^2^ of the original scales with item reduction (RQ1b)					
Item #	Scale	α_Starr et al. 7,_ (*N* = 127)^2^	α_Sherman et al. 9,_ (*N* = 297)^2^	*M*_*orig*_*(SD)/* *M*_*red*_*(SD)*^*3*^	Std factor loadings	*df*	χ^2^	SRMR	CFI	α	Std factor loadings	*df*	χ^2^	SRMR	CFI	α
	**Global teacher identity**	.**86**	.**86**	2.47 (0.89)/2.60 (0.97)		2	9.075*	.**034**	.**961**	.**78**		0	.000***	.**000**	**1.000**	.**86**
1	I see myself as a teacher.			2.88 (1.07)	.782					.72	.801					
2	I would miss teaching if I stopped doing it.			2.14 (1.21)	.811					.70	.815					
3	I truly enjoy the role of a teacher.			2.76 (1.01)	.880					.65	.862					
*4*	*I have looked for opportunities to teach*.^*4*^			2.12 (1.24)	.393					.86	–					
	**Feeling intrinsic satisfaction from teaching**	.**81**	.**80**	2.79 (0.81)		2	6.718*	.**038**	.**975**	.**81**			**Original scale kept**			
5	Working with students and/or residents has its costs, but it’s worth it.			3.11 (0.81)	.550					.81						
6	I find satisfaction watching my students and/or residents’ progress.			2.97 (0.88)	.555					.81						
7	Teaching makes my job more rewarding.			2.66 (1.15)	.915					.97						.
8	It is important to me to work in a teaching practice.			2.42 (1.17)	.859					.72						
	**Having knowledge and skills about teaching**	.62	.60	2.01 (0.64)/2.30 (0.87)		2	10.749**	.**051**	.842	.58		−1	.000***	.**000**	.**964**	.59
9	I feel skilled as a teacher of students and/or residents.			2.12 (1.11)	.676					.29	.737					
*10*	*It is important to develop my teaching skills.*			3.03 (1.03)	.291					.11	–					
11	Students and/or residents regard me as an effective teacher.			2.49 (0.89)	.746					.30	.695					
*12*	*I read journals about medical education, e.g., Academic Medicine.*			0.41 (0.80)	.327					.12	–					
	**Belonging to a group of teachers**	.**88**	.**76**	2.05 (0.84)/2.14 (0.87)		2	19.940***	.**059**	.875	.**77**		0	.000***	.**000**	**1.000**	.**75**
13	I frequently talk to colleagues about teaching.			1.59 (1.07)	.613					.71	.538					.
*14*	*I feel part of a community of teachers.*			1.78 (1.17)	.558					.75	–					
15	It is helpful to be able to discuss the progress of students and/or residents with colleagues.			2.66 (1.00)	.663					.75	.633					
16	I enjoy sharing ideas about teaching.			2.17 (1.12)	.891					.65	.977					
	**Believing that being a doctor means being a teacher**	.51	.58	3.03 (0.57)/3.26 (0.63)		2	**2.461**	.**027**	.**992**	.52		0	.000***	.**000**	**1.000**	.64
17	I do a good job teaching patients about their health.			3.23 (0.73)	.607					.37	.621					
*18*	*I use similar skills to teach patients and students and/or residents.*			2.32 (0.99)	.146					.63	–					
19	I enjoy teaching patients.			2.90 (1.07)	.755					.25	.730					
20	Teaching patients is essential to being a good doctor.			3.63 (0.63)	.511					.45	.527					.
	**Feeling responsibility to teach**	.**78**	.**81**	2.92 (0.74)		2	**2.806**	.**022**	.**993**	.**77**			**Original scale kept**			
21	All physicians have an obligation to teach the next generation of doctors.			3.47 (0.83)	.469					.78						
22	I consider teaching to be a personal responsibility.			2.58 (1.11)	.691					.69						
23	It’s important to contribute to medical education.			3.02 (0.83)	.800					.67						
24	I find it satisfying to think that I am contributing to the profession by teaching.			2.63 (1.07)	.764					.69						
	**Sharing clinical expertise**	.61	.67	2.82 (0.66)/2.54 (0.82)		2	**2.692**	.**030**	.**990**	.63		0	.000***	.**000**	**1.000**	.68
*25*	*Primary care preceptors [Replaced by Physicians] give students and/or residents an important perspective on medicine.*			3.63 (0.62)	.166					.68	–					
26	I am good at teaching students and/or residents to form relationships with patients.			2.59 (1.00)	.921					.39	.955					
27	I teach the importance of developing long-term relationships with patients.			2.45 (1.18)	.558					.55	.541					
28	I am a role model for students and/or residents who want to work in primary [Replaced by *patient*] care.			2.58 (0.96)	.511					.53	.493					
	**Receiving rewards for teaching**	.58	.**79**	2.08 (0.74)/2.50 (0.83)		2	9.183**	.**041**	.792	.50		0	.000***	.**000**	**1.000**	.53
*29*	*The medical school rewards my teaching (e.g., monetary rewards, a parking pass, library privileges).*			0.82 (1.19)	.240					.53	–					
30	Teaching has contributed to my career advancement.			1.76 (1.31)	.516					.34	.450					
31	It is important that the medical school and residency program recognize my teaching in some way.			3.02 (1.11)	.449					.46	.460					
32	I enjoy the recognition I get as a teacher.			2.72 (1.02)	.637					.37	.697					
	**Desired outcomes**	.**76**	.**80**	2.64 (0.79)/2.74 (0.92)		5	21.367***	.149	.**908**	.**75**		0	.000***	.**000**	**1.000**	.**81**
33	I would like to be a more skillful teacher.			2.88 (1.03)	.644					.67	.595					.
34	I would like to be part of a community of teachers.			2.59 (1.13)	.858					.66	.916					
*35*	*I would like to be a better teacher for my patients.*			2.25 (1.19)	.518					.72	–					
36	I would like to spend more time teaching students and/or residents about primary [Replaced by *patient*] care.			2.75 (1.10)	.811					.67	.785					
*37*	*I would like to be rewarded for my teaching.*			2.71 (1.12)	.246					.81	–					

^1^Fit indices in bold are acceptable; ^2^bold Cronbach’s α > .70 considered acceptable; ^3^*N* varies between 143 and 147 due to potential missing responses in various items in the paper-based questionnaires; ^4^Scales which benefited from reduction: items in italic ; * *p* < 0.05; ** *p* < 0.01; *** *p* < 0.001.

We draw the following conclusion: The scales are economically applicable to survey contexts with larger groups but could benefit from further development, which – to the best of our knowledge – neither the instrument’s developers [7] themselves nor other researchers have done so far. Therefore, we postulate the following aim for this study: *Improvement of psychometric properties of a questionnaire instrument that measures physicians’ teacher identity (scales created by [7]) and develop it for applicability to heterogenous survey samples at university hospitals*. Especially at university hospitals, there is a large turnover of employees with varying teaching expertise, wherefore items need to be answerable by heterogeneous samples. Concretely we address the following research questions (RQ):

RQ 1a. Can we confirm the scale structure suggested by Starr et al. [7] in a sample of physicians with varying levels of teaching experience at a university hospital?

RQ 1b. Will reducing the number of items within the scale structure suggested by Starr et al. [7] improve the factorial validity and internal consistency of the scales?

Taking into consideration the challenges previously outlined regarding the psychometric properties of the instruments, we acknowledge that RQ 1a and RQ 1b may not yield satisfactory outcomes for all nine proposed scales. Consequently, we propose the following research inquiries to be tackled through a supplementary exploratory and content-focused methodology.

RQ 2. What scale structure can be revealed by an exploratory factor analysis?

RQ 3a. What scale structure can be retrieved from findings in RQ 1–2 and an additional theoretical item content analysis of the research group?

RQ 3b. Can we confirm the scale structure revealed in RQ 3a?

## Methods

### Questionnaire instrument

The set of 37 items formulated by Starr et al. [7] (for the detailed items, see Table 1 in the Results section) was used in its German version – which had not yet been validated or published. We got the German instrument version from another German research group who had translated the original English items to German, which were in a next step externally retranslated to English. Finally, the original wording and retranslation were compared to optimize the German version (2020 personal information by von Kleist-Retzow, unreferenced) (translation – retranslation procedure cf [10]).

To make the items more relevant to clinical physicians, slight modifications were made to some of the items that were originally developed for primary care. For example, the wording ‘primary care preceptors’ was replaced with ‘physicians.’ Changes are indicated in Table 1. Items were answered on a Likert-scale 0–4 (0 = fully disagree; 4 = fully agree).

### The study’s context

The teacher identity scales were included in a survey targeting all physicians (*N*_May 2020_ = 666) working at a German university hospital. The hospital had been experiencing a transition from a municipal maximum care hospital to a university hospital, assisted by the establishment of a new medical faculty. This organisational change process presumably influenced clinicians’ identification with the (new) role of a teacher. Although the transformation from a care to a university hospital seldom occurs, our sample might be representative of existing university hospitals that undergo reforms in their medical curricula. All physicians, regardless of their current involvement in teaching, were welcome to participate in the survey.

### Sample

In total, *N* = 147 (22% response rate) clinicians gave their informed consent to participate without incentives in either an online (78.9% participants) or printed survey (21.1% participants). In printed questionnaires missing values could not be avoided, online survey enforced an answer for each item. The instruments and consent documents were approved by the data protection supervisor, the hospital’s staff advisory board and the head of the ethics committee of the University of Augsburg, who excluded ethical concerns of any kind (negative clearance certificate issued on 2 March 2020). The study was conducted in accordance with the criteria of the Declaration of Helsinki [11]. The survey questionnaire included additional items on demographics (age, gender), clinical and teaching experience to describe the sample for this validation study.

### Analysis

The dataset was prepared in SPSS 28 [12] by assigning missing values (coded as 9999), followed by generating descriptive statistics (*M, SD* reported in Table 1) for each item and the suggested scales by Starr et al. [7]. The data was then imported in MPlus version 8.8 [13].

RQ 1a. In MPlus, we performed a confirmatory factor analysis (CFA) separately for each of the suggested nine scales by Starr et al. [7], using all 37 items. We evaluated the fit indices, including Chi-square (χ^2^), standardized root means square residual (SRMR), and comparative goodness of fit index (CFI). These indices are considered robust in samples of *N* = 100–200 [14]. For sample sizes ranging from 100 to 200, it is commonly suggested to use a cut-off criterion of χ^2^/df <3 to determine an acceptable fit [14]. SRMR of less than 0.08 are considered ideal [15], a CFI greater than 0.90 [16] reflects a good model fit. Additionally, we calculated standardized factor loadings for each item. Standardized factor loadings greater than .30 were considered acceptable [14].

After conducting the CFAs, we calculated Cronbach’s α for the scales developed by Starr et al. [7]. Furthermore, we report the impact of reducing items on the scales’ Cronbach’s α.

RQ 1b. Based on the standardized factor loadings of each item, fit indices of the scale, and the potential improvement of α through item deletion, we discussed the potential benefits of reducing challenging items. We then performed CFAs on the reduced scales and evaluated α once again.

RQ 2. As anticipated, the outcomes obtained from steps 1a and 1b did not yield an optimal solution. Thus, it became necessary to evaluate RQ2, with the intention of retaining most of the original items. We proceeded with an exploratory factor analysis (EFA) to explore the possibility of uncovering a different underlying structure. In accordance with Castello et al. [17], we used a principal axis factoring with promax rotation on the 37 items. This is a robust method for extracting factors that are likely to be correlated, which is assumed for an instrument measuring the construct teacher identity across various scales [14]. Again, standardized factor loadings greater than .30 were deemed acceptable for this analysis [14].

RQ 3a. We then discussed how scales could be rearranged by integrating confirmatory and exploratory findings and a theoretical item content analysis. The objective was to retain as many items as possible and incorporate insights from the findings of RQ1b to facilitate a beneficial rearrangement. We propose a (partially) new structure for the instrument and suggest reformulating certain items to ensure their future inclusion in the instrument.

RQ 3b. CFAs applying the same fit indices (χ^2^, SRMR, CFI) as in RQ1a and b, as well as Cronbach’s α were applied to investigate the suggested scale structure.

## Results

### Sample description

#### Demographics

Of the respondents, 20.4% were below 30 years old, 49% were between the ages of 30 and 49, and 30.6% were over 50 years old. A slight majority (56.2%) comprised males, 42.5% were females, and 1.2% indicated diverse genders.

#### Clinical experience

Regarding their professional status, 66% were specialists or higher (for example chief physician). Those who worked as full-time clinicians comprised 85%, and 57.8% reported over 10 years of clinical experience, with 36.3% having worked at the studied hospital for more than 10 years.

#### Teaching experience

Among the respondents, 68.3% reported previous teaching experience, with 42% stating more than 10 years of experience. Among those with teaching experience, 46.4% had taught nurses, 61.9% residents, and 95.9% medical students. In total, 42.2% had participated in at least one training course in medical didactics. Up to the point of taking the survey (May 2020), 45.9% of the sample were not actively teaching, 44.5% taught 1–5 hours a week, and approximately 10% taught more than 5 hours. Regarding the new study programme, 52.7% indicated that they were basically informed about the concept, and 18.2% were involved in its conceptualisation. The remaining 29.1% did not know anything about the programme.

### RQ 1a & RQ 1b: Confirmatory identification of factorial valid scales

The findings of the CFAs for the original scales, which included 37 items (RQ1a), as well as the results for the confirmatory factor analysis with item reduction (RQ1b) are presented in Table 1 in the original item order by Starr et al. [7].

#### Confirmed scales

*Feeling intrinsic satisfaction from teaching* and *Feeling responsibility to teach* could be confirmed with good fit indices and good Cronbach’s α of .81 and .77.

#### Scales that benefited from item reduction

Model fit and internal consistency of the *Global teacher identity* scale benefited from removing item #4 (‘I have looked for opportunities to teach.’), which has the lowest factor loading of .393. It is possible that item #4 is too artificial for daily clinical routines, where teaching is a compulsory task within the given curricula.

In *Belonging to a group of teachers*, item #14 (‘I feel part of a community of teachers.’) has an acceptable, but the lowest factor loading of .558. From a theoretical perspective, it is the only item that does not investigate exchange about teaching but rather emotional attachment to a teaching community. From a statistical perspective, removing item #14 improves the model fit and maintains internal consistency as indicated Cronbach’s α of .75.

*Desired outcomes* revealed an acceptable Cronbach’s α of .75 but based on SRMR, we were unable to confirm the scale structure. To improve the model fit, we removed item #37 (‘I would like to be rewarded for my teaching.’) with a poor loading of .246. We also suggest that items related to rewards for teaching could benefit from being consolidated into *one* rewards scale. Additionally, item #35 (‘I would like to be a better teacher for my patients.’), is the only item referring to patients. After reducing items #35 and #37, we obtained excellent fit indices and a Cronbach’s α of .81.

#### Scales with a remaining lack of internal consistency after item reduction

All other scales remained with challenges in internal consistency as indicated by Cronbach’s α. Fit indices indicating factorial validity could be improved after item reduction.

For *Believing being a doctor means being a teacher*, removing item #18 (‘I use similar skills to teach patients and students and/or residents.’), which has a poor factor loading of .146, improves Cronbach’s α to .64, but it is still not at an acceptable level. From a theoretical perspective, #18 is the only item that includes a comparison of teaching patients and students, whereas the other items only refer to patients.

Removing item #25 (‘Primary care preceptors [replaced by *Physicians*] give students and/or residents an important perspective on medicine.’) (low factor loading of .116) from the *Sharing clinical expertise* scale, results in a slight improvement and an almost acceptable Cronbach’s α of .68. Theoretically, item #25 is the only item in the scale that encourages reflection on the role of physicians in sharing expertise, while items #26–28 express a personal reflection (‘I am good…’; ‘I teach…’; ‘I am a role model…’).

In *Having knowledge and skills about teaching*, item #10 (‘It is important to develop my teaching skills.’) with an inappropriate loading of .291 and item #12 (‘I read journals about medical education, e.g., Academic Medicine.’) with a rather low loading of .327 were reduced. Item #12 may not be closely related enough to what physicians do to improve their knowledge about teaching. This becomes also apparent in the low *M*_*#12*_ = 0.41 (*SD* = 0.80) which is in big discrepancy to e.g., *M*_*#10*_ = 3.03 (*SD* = 1.03). Additionally, problematic might be that item #10 represents one’s attitude towards developing teaching skills, while items #9 (‘I feel skilled as a teacher of students and/or residents.’) and #11 (‘Students and/or residents regard me as an effective teacher.’) encourage reflection on teaching experience.

Upon a poor Cronbach’s α of .50 and an unsatisfactory CFA fit indices in *Receiving rewards for teaching*, we tested the scale without item #29 (‘The medical school rewards my teaching (e.g., monetary rewards, a parking pass, library privileges.’) based on the weak factor loading of .240. This resulted in an improvement in fit indices, but Cronbach’s α remained weak at .53. From a theoretical content perspective, items #29, 31, and 32 can only be answered by physicians who have been involved in teaching. Additionally, there are items in the scale that assess a rather extrinsic reward perspective (e.g., #29), while others focus rewards also addressing the intrinsic motivation spectrum (#32 ‘I enjoy the recognition I get as a teacher.’). This mix of motivational factors may lead to inconsistency in the scale, which is also reflected in the discrepant mean values (*M*_*#29*_ = 0.82 *SD* = 1.19; *M*_*#32*_ = 2.72 *SD* = 1.02).

### RQ 2: Exploring scale reassembling

To begin with, we examined the correlation matrix of the 37 items to identify any items that did not correlate with any other item above .30 and those that correlated above .90. Item #25 (‘Primary care preceptors [Replaced by *Physicians*] give students and/or residents an important perspective on medicine.’) did not meet the criteria of having a correlation above .30 with at least one other item. From a statistical standpoint, this suggests that #25 does not contribute to the construct of teacher identity. Additionally, item #25 had displayed poor factor loadings in the confirmatory factor analysis (RQ1a), and its removal improved the fit of the scale related to sharing clinical expertise. Therefore, we removed it. We conducted the principal axis factoring once again with 36 items.

The preconditions for further factor interpretation were met, including the Kaiser-Meyer-Olkin criteria (>.60), a significant Bartlett’s test of sphericity, and anti-image correlations of all items (>.60). Following the Kaiser-Guttman criteria (>1.0), an 8-factor solution was obtained, explaining the distributed variance as follows: 35.9% for factor 1, 8.2% for factor 2, 5.4% for factor 3, and less than 5.0% for factors 4–8. Considering content analysis, factor loadings (as reported in Table 2), and factors explaining more than 5% variance, we identified the following three meaningful scales:

**Table 2.** Results of principal axis factoring (promax rotation) (RQ 2).

		Factors and their explained variance							
Item #	Scale	F 1 35.9%	F 2 8.2%	F 3 5.4%	F 4 <5%	F 5 <5%	F 6 <5%	F 7 <5%	F8 <5%
1	I see myself as a teacher.	.600^1^				.333			
2	I would miss teaching if I stopped doing it.	.736							
3	I truly enjoy the role of a teacher.	.728							
4	I have looked for opportunities to teach.		.809						
5	Working with students and/or residents has its costs, but it’s worth it.	.468							
6	I find satisfaction watching my students and/or residents’ progress.			.355					
7	Teaching makes my job more rewarding.	.721							
8	It is important to me to work in a teaching practice.	.743							
9	I feel skilled as a teacher of students and/or residents.							−.728	
10	It is important to develop my teaching skills.		.688						
11	Students and/or residents regard me as an effective teacher.	.365		.548					
12	I read journals about medical education, e.g., Academic Medicine.				.665				
13	I frequently talk to colleagues about teaching.				.556				
14	I feel part of a community of teachers.	.492			.477				
15	It is helpful to be able to discuss the progress of students and/or residents with colleagues.		.641			−.321			
16	I enjoy sharing ideas about teaching.		.616						
17	I do a good job teaching patients about their health.			.576				−.391	.435
18	I use similar skills to teach patients and students and/or residents.	−.315					.562		
19	I enjoy teaching patients.			.837					.321
20	Teaching patients is essential to being a good doctor.								.812
21	All physicians have an obligation to teach the next generation of doctors.					−.311	.870		
22	I consider teaching to be a personal responsibility.	.453					.482		
23	It’s important to contribute to medical education.	.776							
24	I find it satisfying to think that I am contributing to the profession by teaching.	.742							
*25*	*Primary care preceptors [Replaced by Physicians] give students and/or residents an important perspective on medicine.*								
26	I am good at teaching students and/or residents to form relationships with patients.			.814					
27	I teach the importance of developing long-term relationships with patients.			.587			.332		
28	I am a role model for students and/or residents who want to work in primary [Replaced by *patient*] care.	.567	−.316	.585					
29	The medical school rewards my teaching (e.g., monetary rewards, a parking pass, library privileges).				.900				
30	Teaching has contributed to my career advancement.						.455	.540	
31	It is important that the medical school and residency program recognize my teaching in some way.					.714			
32	I enjoy the recognition I get as a teacher.	.689							
33	I would like to be a more skillful teacher.		.784						
34	I would like to be part of a community of teachers.		.604						
35	I would like to be a better teacher for my patients.		.499					.552	
36	I would like to spend more time teaching students and/or residents about primary [Replaced by *patient*] care.		.718						
37	I would like to be rewarded for my teaching.					.856			

^1^Only standardized factor loadings > .30 are reported for reader friendliness

#### Enjoyment and satisfaction in teaching

This scale, represented by factor 1 which explains the most variance at 35.9%, includes items that express enjoyment and satisfaction derived from teaching. Based on content interpretation and factor loadings, we suggest including items #1, #2, #3, #5, #7, #8, #14, #23, #24, and #32 in this scale.

#### Development and progress of teaching

Factor 2 explaining 8.2% variance encompasses items reflecting the willingness to develop one’s teaching skills and satisfaction derived from observing progress. Considering factor loadings and content analysis, we propose including items #4, #10, #15, #16, #33, #34 #35, and #36 in this scale.

#### Teaching self-concept of ability

Factor 3, accounting for 5.4% explained variance, consists of items that reflect physicians’ self-conviction in teaching (e.g., ‘I am good at…’). We recommend including items #6, #11, #17, #19, #26, #27, and #28 in this scale.

The remaining 11items demonstrated factor loadings on factors 4–8, each explaining less than 5% variance and exhibiting less content consistency.

### RQ 3a & 3b. Suggested reassembled scales and their confirmation

We utilized the confirmatory and exploratory findings from RQ1a, b and RQ2 and conducted a theoretical content analysis for each item. This process resulted in the identification of seven scales and four single items, which are presented in Table 3.

**Table 3.** Suggested reassembled questionnaire instrument to measure physicians’ teacher identity (RQ 3a & b).

				Fit indices^1^ and internal consistency of the reassembled scales				Categorization of items in accordance with van Lankveld et al.’s [1] teacher identity as a continuum		
Item #	Scale	Std factor loadings	*df*	χ^2^	SRMR	CFI	α	Self-conviction	Reflection of context-dependent action	Context-dependent action
	**Feeling intrinsic satisfaction from teaching**		2	6.718*	.**038**	.**975**	.**81**			
5	Working with students and/or residents has its costs, but it’s worth it.	.550						X		
6	I find satisfaction watching my students and/or residents’ progress.	.555							X	
7	Teaching makes my job more rewarding. *Teaching [would] make[s] my job more rewarding*. ^*2*^	.915							X	
8	It is important to me to work in a teaching practice.	.859							X	
	**Feeling responsibility to teach**		5	**4.624**	.**028**	**1.000**	.**75**			
20	Teaching patients is essential to being a good doctor.	.352						X		
21	All physicians have an obligation to teach the next generation of doctors.	.479						X		
22	I consider teaching to be a personal responsibility.	.690						X		
23	It’s important to contribute to medical education.	.802						X		
24	I find it satisfying to think that I am contributing to the profession by teaching.	.758							X	
	**Exchange of teaching experience**		0	.000***	.**000**	**1.000**	.**75**			
13	I frequently talk to colleagues about teaching.	.538								X
15	It is helpful to be able to discuss the progress of students and/or residents with colleagues.	.633							X	
16	I enjoy sharing ideas about teaching.	.977							X	
	**Identification and enjoyment of the teaching role**		9	**15.744**	.**030**	.**980**	.**83**			
1	I see myself as a teacher.	.766						X		
2	I would miss teaching if I stopped doing it.	.766							X	
3	I truly enjoy the role of a teacher.	.921							X	
14	I feel part of a community of teachers.	.637							X	
19	I enjoy teaching patients.	.336							X	
32	I enjoy the recognition I get as a teacher.	.655							X	
	**Development of teaching**		9	41.446***	.**050**	.**909**	.**86**			
4	I have looked for opportunities to teach.	.562								X
10	It is important to develop my teaching skills.	.845						X		
33	I would like to be a more skillful teacher.	.732						X		
34	I would like to be part of a community of teachers.	.796						X		
35	I would like to be a better teacher for my patients.	.567						X		
36	I would like to spend more time teaching students and/or residents about primary [Replaced by *patient*] care.	.790						X		
	**Teaching self-concept of ability**		5	**7.561**	.**038**	.**976**	.**77**			
9	I feel skilled as a teacher of students and/or residents.	.612						X		
11	Students and/or residents regard me as an effective teacher.	.778							X	
17	I do a good job teaching patients about their health.	.483						X		
28	I am a role model for students and/or residents who want to work in primary [Replaced by *patient*] care.	.642						X		
26	I am good at teaching students and/or residents to form relationships with patients.	.672						X		
	**Desired rewards for teaching**		0	.000*	.**000**	**1.000**	.53			
30	Teaching has contributed to my career advancement. *I expect teaching to contribute to my career development*.	.213							X	
31	It is important that the medical school and residency program recognize my teaching in some way.	.974							X	
37	I would like to be rewarded for my teaching.	.546							X	
	**Single items which were excluded from any scale based on CFA, EFA findings and theoretical considerations**									
12	I read journals about medical education, e.g., Academic Medicine.									X
18	I use similar skills to teach patients and students and/or residents.									X
27	I teach the importance of developing long-term relationships with patients.									X
29	The medical school rewards my teaching (e.g., monetary rewards, a parking pass, library privileges). *I expect the medical school to reward my teaching (e.g., monetary rewards, a parking pass, library privileges).*								X	
25	Primary care preceptors [Replaced by *Physicians*] give students and/or residents an important perspective on medicine.									

^1^Fit indices in bold are acceptable; ^2^Items in italics = suggested reformulation for heterogeneous samples; * *p* < 0.05; *** *p* < 0.001.

#### Scales identified on confirmatory findings

We retained *Feeling intrinsic satisfaction from teaching* as originally suggested by Starr et al. [7] as it was confirmed in our sample and revealed a good Cronbach’s α of .81. Also, we kept the originally suggested scale *Feeling responsibility to teach* and added item #20, which, from a theoretical perspective, also expresses a conviction regarding teaching obligations. The scale demonstrates a good model fit and an acceptable Cronbach’s α of .75. Based on the findings of the confirmatory factor analysis in RQ1b, we propose *Exchange of teaching experience* which includes three items related to physicians’ exchange of teaching experience. The scale exhibits an excellent model fit, and its Cronbach’s α of .75 indicates acceptable internal consistency.

#### Scales identified on exploratory findings

*Identification and enjoyment of the teaching role* consists of six items that predominantly loaded on factor 1. These items reflect physicians’ identity and enjoyment in their teaching role. The scale demonstrates a good model fit and internal consistency indicated by Cronbach’s α = .83. *Development of teaching* comprises six items that loaded on factor 2. These items assess physicians’ intention to develop their teaching practice. The scale demonstrates an acceptable model fit and internal consistency (Cronbach’s α = .86). *Teaching self-concept of ability* includes five items that express physicians’ self-concept of their teaching ability. The scale exhibits a good model fit and its Cronbach’s α of .77 indicates internal consistency.

#### Scale identified on theory-based considerations

*Desired rewards for teaching* includes three items assessing physicians’ desired rewards for their teaching. However, the scale still lacks a satisfactory Cronbach’s α. Item #30 is only answerable by physicians already involved in teaching, so we suggest reformulating it.

#### Single items

We excluded five items from any scale to maintain their model fits and internal consistency. We suggest keeping four of them as single items in the instrument.

- Item #12 (‘I read journals about medical education, e.g., Academic Medicine.’): As previously described, this item may not accurately reflect how physicians improve their knowledge about teaching.
- Item #18 (‘I use similar skills to teach patients and students and/or residents.’): This item is only applicable to physicians involved in teaching and might pose problems due to its comparative nature.
- Item #27 (‘I teach the importance of developing long-term relationships with patients.’): This item does not apply to all disciplines in a university hospital, as long-term patient relationships may not be relevant in certain disciplines (e.g., anesthesiology, where a trustworthy first impression is rather crucial).
- Item #29 (‘The medical school rewards my teaching (e.g., monetary rewards, a parking pass, library privileges).’): This item requires reformulation to be answerable for heterogeneous samples. We suggest rephrasing it as ‘I expect the medical school to reward my teaching (e.g., monetary rewards, a parking pass, library privileges).’ From a theoretical perspective, this item could be tested in the *Desired rewards for teaching* scale. In our study, its inclusion in this scale resulted in weak fit indices and an unsatisfactory Cronbach’s α, so we proposed it as a single item.

Item #25 (‘Primary care preceptors [Replaced by *Physicians*] give students and/or residents an important perspective on medicine.’): As shown in the correlation matrix in principal axis factoring, this item does not correlate with any other item above .30, suggesting that it does not represent the construct of teacher identity. We therefore suggest an exclusion of item #25 from the instrument.

## Discussion

In this study, to the best of our knowledge, we have further developed the only existing quantitative questionnaire instrument (in our context in its translated German version) used to measure physicians’ teacher identity [7] on a sample of *N* = 147 clinicians. We intended to contribute to the development and modification of existing instruments to improve their psychometric properties – a research gap identified by Hanna et al. [6].

By employing a confirmatory approach, our goal was to acquire insights into how the current instrument can be enhanced within the proposed scale structure. Through the integration of confirmatory results, additional exploratory findings, and a thorough item content discussion, we proposed a newly optimized scale structure (Table 3).

*First*, recommend that an instrument measuring physicians’ teacher identity be not only answerable by those who are currently involved in teaching, e.g., item #18 (‘I use similar skills to teach patients and students and/or residents.’) does clearly not meet this criterion which became evident in factorial testing. Also, scales should make sense to physicians from any discipline. A broader scope of the instrument can help faculty developers in understanding the teacher identity in novice, advanced and prospective medical teachers from various areas of expertise. Examples include university hospitals with staff fluctuation, where not everyone is always involved in teaching, such as new staff entering residency, as in the study by Sherman et al. [9]; medical teachers of elective subjects offered occasionally; and newcomers from care hospitals without a teaching tradition. Reformulations of certain items, such as item #29 (‘I expect…’) instead of ‘Medical school rewards my teaching’ can accommodate this broader applicability, as the ‘expect’ formulation is answerable by all clinicians, regardless of their current teaching activity or expertise.

*Second*, the *I*-formulation (‘I expect …’) would also meet the definition of teacher identity as a continuum of a person’s self-conviction and a context-dependent action [1] (p. 125). Adopting this teacher identity continuum perspective, we categorized the items in Table 3 according to self-conviction (‘Identity is something *I* have’) and context-dependent action (‘Identity is something *I* do in a context’). It became evident that some items clearly fit into one category or the other. For instance, item #1 (‘I see myself as a teacher.’) reflects self-conviction, while item #13 (‘I frequently talk to colleagues about teaching.’) represents context-dependent action. However, there were items, like #14 (‘I feel part of a community of teachers.’) – which from our perspective – rather represent the *reflection of a context-dependent action*. Some of the identified scales tend to include items that reflect self-conviction, such as the scale *Development of teaching* with its many ‘I would like…’ formulations. On the other hand, the scale *Identification and enjoyment of the teaching* role primarily consists of items that express reflections of context-dependent action. As shown in Table 3, the validation of the instrument resulted in scales that are relatively consistent in terms of the teacher identity continuum. A lack of this consistency in some scales of the original instrument may explain the originally unsatisfactory low Cronbach’s α. Furthermore, it was evident that the four items we suggest as single items primarily represent context-dependent actions, thereby facilitating easier responses from individuals involved in teaching.

*Third*, by combining a confirmatory and exploratory approach with a critical theoretical item analysis, we could identify seven scales which at least in our sample revealed acceptable to good model fits. Despite the exploratory approach suggesting a simplified therefore more economic scale structure with fewer items, we opted to prioritize the more differentiated seven scale structure. Reasons were 1) the objective to retain as many items as possible within scales rather than keeping them as single items; 2) adhering to the logic of Starr et al. [7,8], who posited that teacher identity encompasses various facets as identified in their interview data, which underpinned their scale development and 3) mixed-method approaches can be a promising way to improve instruments’ content validity – which is still under-researched [18]. Koller et al. [18] (p. 6) suggest – besides psychometric testing – challenging items with experts on the questions ‘Do you think the item could be difficult to understand? If yes, why?’ and ‘Do you think the item might have a different meaning for certain groups of people (e.g., men vs. women, younger vs. older participants, participants from different professional fields, or levels of education)? If yes, why?’. We recommend developing the instrument further by a) validating our scale structure in samples of respondents with varying levels of expertise and experience in teaching (which reflects the reality of a university hospital) and b) ask both experts, but also potential addresses on items understandability and perception of meaning. Items such as #23 (‘It’s important to contribute to medical education.’) might leave more room for interpretation especially for those who are not yet involved in medical education.

## Limitations

Our sample is restricted in its size, response rate and single centre in a transformation process. Additionally, we validated a German translation of the original instrument developed by Starr et al. [7], which requires further approval in the English-speaking context to check for potential cultural interpretations and relevance of language nuances. The 22% of the *N* = 666 physicians participating in the survey were probably (and as suggested by our positive mean values) well-disposed toward the topic of teaching. As stated in the sample description, approximately 50% of the responding clinicians reported being informed about the new study programme, and almost 20% were even involved in its conceptualisation and were thus probably willing to accept an email invitation to a survey on teaching. Additionally, the applied scales might have enforced social desirability in the clinicians’ answering behaviour. Given the transformation to a university hospital, the clinicians might have assumed that a positive identity toward teaching would be expected.

## Data Availability

The data that support the findings of this study are not openly available due to reasons of sensitivity and are available from the corresponding author upon reasonable request.
